# To move without moving: a perspective article on motor imagery

**DOI:** 10.3389/fpsyg.2025.1697086

**Published:** 2025-12-09

**Authors:** Pedro Duarte-Mendes, André Ramalho, Maurizio Bertollo, Henrique Pereira Neiva, Daniel Almeida Marinho

**Affiliations:** 1 School of Education, Polytechnic Institute of Castelo Branco, Castelo Branco, Portugal; 2 Sport Physical Activity and Health Research & Innovation Center (SPRINT), Castelo Branco, Portugal; 3 PSYLAB – Faculty of Human Kinetics, University of Lisbon, Lisboa, Portugal; 4 Department of Medicine and Aging Sciences, BIND-Behavioral Imaging and Neural Dynamics Center, University G. d’Annunzio of Chieti-Pescara, Chieti, Italy; 5 Department of Sport Sciences, University of Beira Interior, Covilhã, Portugal; 6 Research Center in Sports Sciences, Health Sciences and Human Development (CIDESD), Convento de Santo António, Covilhã, Portugal

**Keywords:** movement science, neuroplasticity, mental simulation, cognitive rehabilitation, embodiment

## Abstract

Motor imagery – the mental simulation of movement without execution – activates motor networks with near-physical fidelity. Once considered ancillary, it is now central to neuroplasticity, enhancing skill acquisition, accelerating rehabilitation, and sustaining motor function across the lifespan. From stroke recovery to elite performance, motor imagery demonstrates that movement begins in cognition. As neurofeedback, brain–computer interfaces and virtual reality integrate with mental rehearsal, the boundary between thought and action becomes narrower. This perspective argues that motor imagery is not a cognitive accessory but the neurocognitive foundation of movement – a rehearsal mechanism through which the brain reshapes the body. In doing so, it supports the view that action is cognitively prepared before it is expressed.

## 1 Introduction to motor imagery

Although interrelated, motor imagery (MI), action observation, and mental simulation differ in how they activate internal representations: MI entails the mental execution of movement without physical enactment; action observation involves visually perceiving others’ movements, engaging the motor system via mirror neurons; and mental simulation encompasses both, extending to sensory and emotional components associated with the action (Eaves et al., 2016). Movement can be initiated at the cognitive level before any muscular contraction occurs. In this sense, we argue that MI is not merely a reflection of movement, but its cognitive genesis — a neurocognitive process that enables action, reshapes the motor system, and blurs the boundary between thinking and doing. MI, the act of mentally simulating motion without physical execution (Mulder, 2007), has evolved from a psychological curiosity to a neuroscientific keystone. Functional magnetic resonance imaging (fMRI) studies show that imagining a sprint or a piano chord activates neural networks nearly identical to those engaged during execution (Jeannerod, 2001; Meister et al., 2004).

In a seminal study, Pascual-Leone et al. (1995) concluded that. That MI can modulate the central motor system and may therefore, be used to support motor learning, to maintain motor abilities in temporarily immobilized patients, and to complement rehabilitation. Although much research has distinguished motor imagery from actual movement, recent theories suggest that athletes can engage in a more dynamic form of imagery by assuming congruent body positions and incorporating spatial or temporal aspects of the movement without fully executing (Guillot et al., 2021) it and can be useful also combining action observation (Wright et al., 2014).

Across developmental and clinical contexts, MI appears to support the acquisition, maintenance, and restoration of motor function (Duarte-Mendes et al., 2025). Children with developmental coordination disorder (DCD) benefit from MI-based interventions that bypass impaired motor planning through alternative neural routes (Ruffino et al., 2017). In sports, MI reduces error rates and enhances consistency, while in stroke rehabilitation, it supports the reactivation of dormant motor circuits (Sharma et al., 2006; Simonsmeier et al., 2018). Even in neurodegenerative conditions such as dementia, structured MI practice has been associated with improvements in cognitive function, affect regulation, and daily functioning (Christakou et al., 2024). These examples underscore MI as a manifestation of the brain’s anticipatory architecture. Moreover, a recent study highlights the different neural and cognitive requirements of fast versus slow motor imagery, offering insights into the specialized functions of the underlying motor and sensory cortex (Sani et al., 2025).

Crucially, MI is not merely symbolic. It engages neural substrates in ways that suggest functional consequences. While it may not substitute physical training, it modifies the conditions under which action becomes possible (Lebon et al., 2018). Neuroprosthetic devices now translate imagined gestures into robotic commands, enabling individuals with paralysis to perform goal-directed tasks through thought-driven interfaces (Bouton et al., 2016).

These findings raise a core question: if imagined acts leave neural and physiological traces, is the boundary between intention and execution a biological illusion?

This article argues that MI is not a supplement, but a neurocognitive substrate of action. The body does not act in isolation. We propose that motor behavior is continuously shaped and reshaped by anticipatory cognitive processes (Frank et al., 2024a; Frank et al., 2024b).

## 2 Neural mechanisms and plasticity in motor imagery

MI is not passive reflection, but rehearsal without motion. Neuroimaging consistently shows that mental simulation recruits the premotor cortex, supplementary motor area, and posterior parietal regions, mirroring the neural architecture of physical execution (Jeannerod, 2001; Meister et al., 2004). Transcranial magnetic stimulation further confirms that MI increases corticospinal excitability, priming motor pathways without muscle contraction (Kitamura and Kamibayashi, 2024; Wright et al., 2014).

When combined with physical training, MI accelerates motor learning more effectively than physical practice alone (Schuster et al., 2011). In clinical populations, particularly individuals with Parkinson’s disease, MI has been shown to enhance motor function, mobility, and daily performance, even when movement is limited (Abraham et al., 2021; Cuomo et al., 2022). Taken together, these studies indicate a substantial overlap between physical and mental forms of motor engagement. In this study, physical and mental effort are viewed as complementary dimensions of motor engagement: physical effort involves physiological and biomechanical exertion during overt movement, whereas mental effort reflects cognitive and neural activation during MI or action observation. Neuroimaging research shows that both forms of effort recruit overlapping motor regions, including the premotor cortex, supplementary motor area, and parietal cortex (Decety, 1996; Jeannerod, 2001; Hardwick et al., 2018), indicating that mental simulation can reproduce aspects of physical execution. Evidence from imagery-based interventions in individuals with neurological diseases further demonstrates that intention and cognitive engagement can induce neural adaptations typically associated with physical practice (Guillot et al., 2008; Heremans et al., 2012), supporting the notion that motor learning can arise through both overt and covert forms of practice, sustaining the view that intention can, in some contexts, act as a relevant driver of neural adaptation. This does not imply that intention always replaces movement, but that it can contribute to the same functional systems.

MI supports plasticity through synaptic tagging and offline consolidation. Neuroplastic changes occur not only during practice, but also during rest periods following imagery sessions (Di Rienzo et al., 2023; Ruffino et al., 2017). Moreover, individual MI capacity correlates with structural and functional brain features: individuals with greater imagery control exhibit increased white matter volume in frontoparietal tracts and stronger connectivity between motor, visual, and somatosensory regions (Furuta et al., 2024).

In stroke rehabilitation, MI combined with physiotherapy improves gait parameters and lower limb strength (Bovonsunthonchai et al., 2020). In expert pianists, MI has been shown to preserve technical fluency during periods of forced rest, maintaining performance through cognitive simulation (Christakou et al., 2021). These examples position MI as an alternative route to skill retention and recovery, especially in contexts where overt movement is compromised.

From an evolutionary standpoint, MI provides a mechanism for anticipatory learning. It enables individuals to simulate risk-heavy or effort-intensive actions internally before committing to them externally. In children with DCD, MI training is linked to improved motor planning and execution, potentially through enhanced white matter integrity in sensorimotor networks (Barhoun et al., 2019). In older adults, MI has demonstrated efficacy in improving balance and mobility, offering a non-invasive intervention where physical training may not be feasible (Nicholson et al., 2019).

MI is not a passive by-product of consciousness but an active rehearsal system embedded in motor cognition. It is preparatory, adaptive, and neurophysiologically consequential. These neural findings provide the foundational basis for the functional applications of MI within the domains of rehabilitation and performance enhancement.

## 3 Motor imagery in rehabilitation and performance contexts

MI is not limited to recovery support or performance refinement – it restructures how the brain prepares for action and adapts to disruption. In stroke rehabilitation, MI has consistently demonstrated effectiveness as a complementary intervention, particularly when integrated into physiotherapy protocols. Improvements have been reported in upper and lower limb function, balance, and gait (Prasomsri et al., 2024). In Parkinson’s disease, MI enhances motor function, mobility, and quality of life, even when basal ganglia circuits are compromised (Michel et al., 2024). These outcomes reinforce the hypothesis that internal simulation can compensate for impaired motor execution by reactivating dormant or underutilized neural pathways.

In high-performance sport, MI is used proactively to refine execution before movement begins. Athletes mentally rehearse techniques to improve precision, strength, and error detection. When structured through the PETTLEP model, MI has been shown to enhance performance across various sports, especially when paired with physical training (Morone et al., 2022). This effectiveness is due to the functional overlap between imagined and executed actions – both activate the motor cortex, cerebellum, and associated sensory regions (Mizuguchi et al., 2012). Additionally, MI engages the brain’s internal forward models, allowing athletes to anticipate the sensory consequences of actions in the absence of movement (Kilteni et al., 2018). Moreover, in an elite athlete, Budnik-Przybylska et al. (2021) showed that guided imagery might induce higher high alpha cortical activity (selective attention) compared to self-produced imagery that enhance low alpha (associated with relaxation).

The convergence of MI and neurofeedback introduces complex ethical and regulatory questions. Electroencephalography-based neurofeedback enables users to self-regulate brain activity in real time, potentially enhancing focus, emotional control, and motor precision (Chang et al., 2011). Though not classified as doping, ongoing debates concern competitive fairness, cognitive enhancement, and accessibility (Corrado et al., 2024). Cultural perspectives further complicate the issue: Western emphasis on visible exertion contrasts with Eastern traditions of internal mastery, reflected in imaging studies showing reduced prefrontal activation in experienced archers (Chang et al., 2011). A recent review by Tosti et al. (2024) underscores neurofeedback’s efficacy in improving performance across multiple domains, suggesting it may become increasingly central in competitive contexts. As mental training becomes increasingly digital, the line between self-mastery and algorithmic aid continues to blur – inviting deeper philosophical inquiry.

However, access to MI technologies is uneven. Systems like BrainGate allow individuals with paralysis to perform tasks via cognitive intent – such as typing, browsing the web, or manipulating digital interfaces (Nuyujukian et al., 2018). Intracortical brain–computer interfaces (BCIs) have enabled users with tetraplegia to interact with commercial tablets and communicate in real time, solely through MI. Yet these advances remain restricted to high-resource research centres. As Müller and Rotter (2017) note, even non-invasive BCIs require technological infrastructure and expertise often unavailable in low- and middle-income settings. While the capacity for MImay be universal, the capacity to train and apply it is not.

Emerging technologies amplify this divide. As virtual reality–MI platforms evolve, the tension between imagined fluency and physical constraint becomes more pronounced. In individuals with advanced neurodegenerative conditions, task complexity in virtual environments may exceed biological feasibility, producing a phenomenon known as embodiment dissonance – a disconnect between the simulated self and the biological body (Kashif et al., 2022). This may lead not only to performance limitations but also to psychological estrangement.

From childhood through aging, MI supports neurofunctional adaptability. Children with DCD benefit from MI by improving cerebellar compensation mechanisms (Ruffino et al., 2017). In older adults, MI helps preserve corticospinal function and mobility, providing continuity where physical capacity begins to decline (Clark et al., 2014). In this sense, MI is not a technique but a form of cognitive continuity – a scaffold for motor capacity that persists even as biological resources diminish.

At its foundation, MI demonstrates a consistent principle: overt movement can be prepared and supported by covert motor processes. Neural rehearsal redefines what the body can do by anticipating what the mind imagines.

## 4 Motor imagery and emerging technologies in neurorehabilitation

MI is no longer confined to internal rehearsal. It is becoming an operational mechanism for embodied interaction in clinical, technological, and rehabilitative contexts. In 2016, a tetraplegic individual used a BCI to drink coffee by translating imagined hand movements into robotic action, bypassing the spinal cord (Bouton et al., 2016). Systems like BrainGate now enable individuals with paralysis to type, grasp objects, and control assistive devices through MI alone, shifting agency from biomechanical function to cognitive intent (Nuyujukian et al., 2018).

Virtual reality (VR) enhances this transformation by creating environments where MI can be paired with visual and sensory feedback. In patients with phantom limb pain, training with virtual avatars activates dormant motor areas and reduces pain intensity, improving prosthetic embodiment and sensorimotor integration (Ortiz-Catalan et al., 2016).

While MI has long been considered a mental rehearsal of movement, recent developments suggest that, when coupled with sensory scaffolding, it becomes a tool of neuromodulatory precision. Platforms that integrate MI with robotic feedback no longer aim merely at symbolic engagement, but at cortical reconfiguration. Evidence from brain-computer interface programs shows measurable improvements in motor function and neural activation in stroke patients (Ma et al., 2024), while the very nature of the feedback - visual, auditory, proprioceptive – has been shown to modulate motor cortical oscillations, shaping the quality and depth of the imagined act (Vukelić and Gharabaghi, 2015). In this sense, imagined movement, when properly supported, moves from representation to intervention.

MI is increasingly incorporated into clinical guidelines. Structured mental rehearsal is now prescribed in stroke rehabilitation programs as a cognitive complement to physical therapy (Bovonsunthonchai et al., 2020). In Parkinson’s disease, clinical trials indicate that targeted MI training improves upper limb function, self-efficacy, and functional performance (Michel et al., 2024). Additional studies show that MI increases corticospinal excitability and enhances cognitive precision, reinforcing its efficacy across behavioral and neurophysiological domains (Anil et al., 2021). While wearable neurofeedback systems remain in development, the premise that thought alone can recalibrate disrupted motor circuits is no longer speculative – it is empirically supported.

However, these advancements introduce ethical considerations. Some users of virtual reality-motor imagery platforms report embodiment dissonance – a sense of disconnection between virtual representations and their physical selves. Philosophical critiques refer to this phenomenon as somatic alienation: the body becomes a programmable interface, more functional than experiential. Concerns have also been raised regarding invasive BCI development, particularly in the context of Neuralink’s primate studies, which have drawn ethical scrutiny related to animal welfare and transparency (Musk and Neuralink, 2019).

MI’s convergence with artificial intelligence and immersive technology raises a critical question: is the body a static biological system, or a modifiable construct shaped by cognitive rehearsal?

As the distinction between real and virtual effort becomes increasingly permeable, the neurocognitive mechanisms underlying movement are no longer secondary. They constitute the primary architecture through which action is imagined, prepared, and potentially executed – redefining the limits of human embodiment.

## 5 Conceptual implications of motor imagery for agency and embodiment

This is the quiet revolution of MI: movement begins not in muscle, but in the synaptic activity that anticipates it. What initiates action is rarely visible.

MI is not merely a technique – it is a primary language of human agency. From Pascual-Leone’s findings on cortical plasticity to BCIs that translate thought into motion (Bouton et al., 2016), MI reveals an evolutionary truth: the brain simulates action as a form of preparation, long before execution. We are not moved solely by muscle, but by internal rehearsals – silent, predictive, and adaptive.

Yet the expansion of MI brings paradox. Neuroprosthetics translate intention into movement; virtual avatars respond to imagined command. As embodiment becomes increasingly programmable, a new tension emerges: between enhanced capacity and disconnection from the physical self. Reports of embodiment dissonance, both in animal models and in human users of immersive technologies, signal a potential estrangement – where the simulation outpaces the substance.

MI leaves us with a critical question: if movement can be generated, refined, and expressed through thought alone, what role remains for the biological body? The answer may lie not in anatomical structure, but in cognitive potential. The body is no longer merely a vessel – it becomes a narrative shaped by intention, a draft in continuous revision.

In the end, MI does not abandon the body – it reframes it. The effort exerted is not only muscular, but mental. The goals reached are not just physical, but neurological. To move, in this new paradigm, is to think dynamically – to treat the imagined as a legitimate pathway to the real. MI shifts the terrain of performance, recovery, and identity. It suggests that, in some contexts, the limit is not motor capacity but the absence of structured cognitive rehearsal.

## Data Availability

The original contributions presented in the study are included in the article/supplementary material, further inquiries can be directed to the corresponding author.
